# The Principles and Practice of Modern Surgery

**Published:** 1849-01

**Authors:** 


### ARTICLE VI.

*The Principles and Practice of Modern Surgery.* By ROBERT DRUITT, Fellow of the Royal College of Surgeons. "Id potissimum agrus ut omissis hypothesibus in praxi nihil adstruat quod multiplici experientia non sit roboratum."—*Act. Erud. Sips.* 1722. A new American from the last London edition. Edited by F. W. SARGENT, M. D., author of Minor Surgery, etc.; illustrated with one hundred and ninety-three wood engravings, pp. 576. Philadelphia, Lea & Blanchard, 1848. (From the publisher, and for sale by J. Keene & Bro., Chicago.)

This is the best work of its size, on the subject of surgery, that has made its appearance on our desk.

For the use of the general practitioner, it may be preferable to many of the larger works, as it has the important facts he wants, in a more condensed form, from which he can get his information with less labor and time, if not with clearer views of the subject.

